# Annotations

**Published:** 1889-01-05

**Authors:** 


### ANNOTATIONS.

The Sweating of Nurses.

THERE have been a sufficient number of letters in one of our daily contemporaries to justify the conclusion that a good many people entertain decided opinions on the question of the overwork of sick nurses. We have had opportunities of examining the recent rules of several hospitals, and they all show that at least the hours of work of a hospital nurse are decidedly "long." It must be surmised that at such centres of medical "light and leading" as hospitals the elementary rules of health will be carried out with exactness and completeness. If physicians and surgeons have any faith at all in their own principles, we cannot conceive it possible that they can allow hundreds—nay, thousands—of young women, many of them of the highest excellence of character, to run the risk of destroying their constitutions. It cannot be anything but an unpardonable absurdity, if it be really true, that healthy women are compelled by hospital rules to make themselves ill in order that they may make unhealthy people well. And that being so, we will not, without more evidence, believe the outcry to be very extensively true, if indeed it be true at all. But the examination of rules already spoken of does, as we have stated, reveal the fact that at certain hospitals the hours of work are many. The rising is early, very early; the time for meals is short; the hours off duty are not adequate; and the retiring time is late. Of course, much depends upon the severity of the duty in estimating the length of time a nurse should be engaged daily; and as sick wards cannot be left, even for an hour, without attendants, it is imperative that some day nurses should be detained very late. But they need not all be kept late at work. It should be possible to arrange relays of attendants, so that none should be employed longer than is compatible with perfect health. We may hope that if the hardships complained of by a few are at all general, the complaints will also become general. If, on the other hand, the cases are exceptional, it is desirable that steps be taken to inform the public on the subject. In any case we insist that the health of nurses ought to be cared for with the utmost solicitude and scientific watchfulness. All such absurd ideas as discipline for the sake of discipline have long been exploded in the great world. They surely do not survive in hospitals. Training for the sake of efficiency, routine for the sake of exactness and aptitude—these we can understand. But rules without reason, authority without purpose, the exaction of unquestioning obedience to orders that disgust by their inconsequent triviality—these are worthy only of the very worst kind of conventual discipline, and ought to find no place at all in any modern hospital. Medical charities are, or ought to be, the abodes of true science. Always and everywhere true science and sound reason are twin sisters. It would indeed be an anticlimax if from these abodes reason has been banished in order to give place to small pedantries and ridiculous autocracies.

The Eating-house Trick.

To the ruses of beggars may be added what should be termed the "eating-house trick" There is near Aldgate station an eating-house, not exactly of a gorgeous or scrupulously-clean description; but from it there issue odours, savoury if somewhat strong, of sausages, pea soup, or other dainties of the "filling at the price" order. A few days ago a hungry man might have been observed gazing with the fascination born of hopeless longing at the rich heaps of polonies, the crisply fried onions, and savoury Irish stew that could be seen through the window. His face was lean and earnest; his clothes were of ragged broadcloth, the cast-off garments of some bulkier man; his boots were slit across the toes, and, practically, non-existent at the heel, so that the spectator saw more than is usual in conventional circles of a pair of broad red feet. The spectator in this case was a member of THE HOSPITAL staff, who, being human and having recently breakfasted, could not look without emotion on a fellow-mortal gazing hungrily into a cook-shop. Therefore he forgot the rules of political economy for the moment, presented the cadaverous gazer with the sum necessary to purchase a modest meal of sausage and mashed potato, and crossed the road, as the hungry one moved with a slow and shuffling pace towards the door of the eating-house. Arrived on the other side of the broad street the hospitaller turned and looked back just in time to see the hungry one turn back from the shop door without entering, and again take up his station in front of the window, studying the polonies with the same earnest gaze. Alas for poor human nature! Is it not possible for any of us to yield to a natural impulse of pity without the risk of encouraging some ingenious rogue? Apparently not. The pursuit of charity requires more careful investigation than the deciphering of Chaldean hieroglyphics. It seems only too probable that the hungry one of Aldgate was a mendicant of the first water, able to entice coins from the most carefully guarded pockets. Had he cared to employ his talents in another way he might have won a great success in melodrama, as the miserable being, father to the heroine whom the gentlemanly swindler of the piece had ruined. He would have required no make-up.

Suicide and Insanity.

WE appear to have made up our minds in this country that all suicides are insane. It is so rare as almost to be impossible to find a coroner's jury giving any other verdict than this of any self-murderer: "Suicide whilst of unsound mind." Nevertheless, it is quite true that in many cases the only single fact in favour of insanity is the suicide itself. So that we are practically coming to this: That suicide alone is a sufficient proof of insanity; that every person who kills himself wilfully is *ipso facto* proved an insane person. If we are content with such a conclusion it would be well to give the idea some more formal and statutory sanction. It seems a waste both of time and money in many cases of suicide to call a jury together for the mere purpose of pronouncing a verdict which is a foregone conclusion. In every instance where there is sufficient evidence to demonstrate the undoubted fact of suicide it ought to be enough to make the demonstration before an ordinary magistrate, or even before the registrar, by means of a regular medical certificate. But is it really the case that every suicide is insane? And if it be, are we to accept the united wisdom of coroner's juries as conclusive on the point? It cannot but be known generally among men of the rank of legislators that the opinion into which we have drifted is by no means universal. Every public schoolboy must be familiar with the fact that many of the thinkers of times not very remote not only disbelieved in the "insane" theory of suicide, but actually argued that, under certain circumstances, suicide was not merely allowable, but honourable and wise—in some cases indeed the only thing to be done. All voluntary martyrs for religion or other causes may be described in a sense as suicides, in that they prefer and choose death rather than the giving up of their own peculiar views. Every one will admit that a woman should prefer death to dishonour. No one would think of describing as insane any woman who should jump into a raging sea if that were her only alternative. Are we so destitute of imagination as to see no other cause for suicide than insanity? The two letters written by Jane Norris, of Canonbury, aged 43—one to her son Bertie, and the other to her daughter Eliza—just before her self-destruction by carbolic acid the other day, show no sign whatever of insanity. The woman was beaten in the struggle with fate; she had no money and no friends. One had refused to take her in; another had apparently let her see that she was an unwelcome burden. What could she do? She might have gone "upon the streets." Would that have been an evidence of sanity? We think not. She certainly did well to prefer death to that; even death by her own hand. The truth is that in London and other large towns the grip of a relentless destiny upon those who fail in the battle of life is overpowering. They begin to go down; and, as if impelled by an enclosing army of pitiless fiends, they feel that no power, human or divine, can avert their doom. Then they despair. A desperate person may be as calm as a stone; and may reason with all the severe logic of a Thomson or a Mill. The syllogism is very simple: "If I live I die; if I die I die. Better die now by a short and effective process, than endure months or years of abject and helpless poverty, disgrace, degradation, misery, and then die." Where is the insanity? Not in the suicide; but in the great and wealthy state that provides no "Bureau for the despairing," where they may at least find guidance and uplifting until the present resistless pressure is past. Some new, less rigidly mechanical, and more human and rational method of dealing with the beaten soldiers who fall out of the ranks of life's army, is called for with painful urgency. Is improvement in this direction quite impossible? If it be this country never confessed to a more shameful impossibility.

